# Pegylated Interferon Alpha-2b in Patients With Polycythemia Vera and Essential Thrombocythemia in the Real World

**DOI:** 10.3389/fonc.2021.797825

**Published:** 2021-12-21

**Authors:** Yingxin Sun, Yifeng Cai, Jiannong Cen, Mingqing Zhu, Jinlan Pan, Qian Wang, Depei Wu, Suning Chen

**Affiliations:** ^1^ Department of Hematology, First Affiliated Hospital of Soochow University, Soochow University, Jiangsu Institute of Hematology, National Clinical Research Center for Hematologic Diseases, Suzhou, China; ^2^ Department of Thrombosis and Hemostasis, Key Laboratory of Thrombosis and Hemostasis of Ministry of Health, Suzhou, China; ^3^ Department of Hematology, The Affiliated Hospital of Nantong University, Nantong University, Nantong, China

**Keywords:** polycythemia vera, essential thrombocythemia, hematological response, molecular response, myeloproliferative neoplasms, pegylated interferon alpha-2b

## Abstract

Several clinical trials have shown promising efficacy of pegylated interferon (Peg-IFN) in the first- and second-line polycythemia vera (PV) and essential thrombocythemia (ET). However, the efficacy and safety of Peg-IFN in the real world have rarely been reported. Hence, we conducted a prospective, single-center, single-arm, open exploratory study, which aimed to explore the hematologic response, molecular response, safety, and tolerability of patients with PV and ET treated with Peg-IFN in the real world. This study included newly diagnosed or previously treated patients with PV and ET, aged 18 years or older, admitted to the Department of Hematology of the First Affiliated Hospital of Soochow University from November 2017 to October 2019. The results revealed that complete hematological response (CHR) was achieved in 66.7% of patients with PV and 76.2% of patients with ET, and the molecular response was obtained in 38.5% of patients with PV and 50% of patients with ET after 48 weeks of Peg-IFN treatment. Peg-IFN is safe, effective and well tolerated in most patients. In the entire cohort, 4 patients (9.1%) discontinued treatment due to drug-related toxicity. In conclusion, Peg-IFN is a promising strategy in myeloproliferative neoplasms (MPNs), and Peg-IFN alone or in combination with other drugs should be further explored to reduce treatment-related toxicity and improve tolerability.

## Introduction

MPNs are clonal hematological malignancies that arise from mutations in the hematopoietic stem cells (HSCs) compartment (1). The two classical BCR-ABL-negative MPNs, PV and ET, are characterized by overproduction of mature blood elements, tendencies toward increased risks of thrombosis and hemorrhage, extramedullary hemopoiesis, and transformation to myelofibrosis or acute myeloid leukemia (1). Janus Kinase 2 (JAK2) V617F is the most common genetic mutation in these diseases and is found in 95% of patients with PV and 50% – 60% of patients with ET, which results in constitutive kinase activity promoting both hematopoietic cells proliferation and a proinflammatory state (2–5). Therapeutic approaches to these diseases have predominantly addressed thrombosis and hemorrhage with cytoreductive therapy, as well as aspirin. Hydroxyurea (HU) has been most frequently used for this purpose and is generally accepted as a frontline therapy for high-risk patients with PV and ET. However, resistance and/or intolerance are common in patients receiving HU, which signifies a poor prognosis, increased mortality, and increased rate of transformation to more advanced MPNs (6). Moreover, HU did not prolong survival, and did not reduce thrombotic and bleeding risks in MPNs patients, even if the patients achieved molecular or hematologic responses (6). Therefore, other drugs that can replace HU are urgently needed. IFN has been used to treat MPNs for more than 30 years. Some early studies have confirmed that recombinant IFN is valuable in patients with classical MPNs. Unfortunately, treatment suspension occurs frequently due to adverse reactions (7, 8). However, IFN can eliminate JAK2-mutated long-term HSCs in some patients, leading to the possibility of cure (9–13). At the same time, a research conducted by Massaro et al. confirmed that IFN can eliminate malignant clones by means of a selective effect on bone-marrow transformed cells in PV (14). Given these advantages of IFN, researchers’ enthusiasm for it never waned. But its widespread use has been limited due to its toxicity and need for frequent use of conventional formulations. As a result, a high proportion of patients discontinue treatment (5, 15–17). The emergence of Pegylated interferon (Peg-IFN) fills this deficiency. Peg-IFN has a longer half-life than conventional IFN, resulting in less frequency of administration and improved tolerability, indicating that Peg-IFN is more suitable for long-term applications than conventional formulations. Several clinical trials have shown exciting effects of Peg-IFN in the first- and second-line PV and ET treatments (15, 18, 19). Silver et al. preferred Peg-IFN for patients who were not eligible for clinical trials, based on the biological properties of Peg-IFN that eliminates mutated long-term HSCs in MPNs (16, 20). At present, the 2021 National Comprehensive Cancer Network (NCCN) guidelines recommend that Peg-IFN should be considered for younger patients in need of cytoreductive therapy. It can be seen that treatment strategies for MPNs are changing, and Peg-IFN plays an important role in MPNs and has been able to replace HU. Nevertheless, the efficacy and safety of Peg-IFN in the real-world setting have rarely been reported. Consequently, we carried on the study to report the hematologic response, molecular response, safety, and tolerability of patients with PV and ET treated with Peg-IFN in the real life.

## Materials and Methods

### Study Design and Participants

This prospective, single-center, single-arm, open exploratory study analyzed the efficacy of Peg-IFN-alpha-2b (Peg-IFN-a-2b; PegBeron, Y shape, 40 kDa, Xiamen Amoytop Biotech, China) in patients with PV and ET. Patients aged 18 years or older with newly diagnosed or previously treated PV and ET who were admitted to the Department of Hematology of the First Affiliated Hospital of Soochow University from November 2017 to October 2019, were included according to the 2008 World Health Organization criteria (21). Other inclusion criteria included the following: no prior use of Peg-IFN, noncomplete response to previous treatment, Eastern Cooperative Oncology Group ≤ 2, serum creatinine < 2.0 mg/dL, serum bilirubin ≤ 2 times the normal upper limit, and voluntary informed consent for participation. The exclusion criteria included allergic to active ingredients of IFN or other components, autoimmune chronic hepatitis, severe liver dysfunction or decompensated cirrhosis, history of severe heart disease (including 6 months with unstable or uncontrolled heart disease), a serious mental disease or a history of severe mental illness, pregnant or planning a pregnancy and lactating women, or men with fertility program, patients with severe kidney diseases (on hemodialysis), epilepsy (requiring anticonvulsive therapy), a history of other malignancies within 3 years, systemic infections (such as hepatitis B/C or human immunodeficiency virus), and those determined as unsuitable by the investigator for this clinical research.

### Procedures

Peg-IFN-a-2b (180 µg) was administered subcutaneously once per week. During treatment, the dose was modified by the treating physician on the basis of toxic effects or absence of drug activity. All adverse events were graded according to the National Cancer Institute Common Toxicity Criteria for Adverse Events (version 2.0). Criteria for discontinuation included clearly documented disease progression (i.e., increased transfusion requirement, splenomegaly, platelet or white blood cell counts more than cutoffs, increased frequency of phlebotomy, or incident thromboembolic events) or absence of clinical response within 6 months of the start of the therapy. Physical assessment and blood counts were measured every 3 months. Bone marrow aspiration, biopsy, and cytogenetics were conducted before treatment. Peripheral blood JAK2 Val617Phe quantification was performed before treatment and every 3 months thereafter.

The primary endpoint was the proportion of patients with a clinical hematological response at 48 weeks of treatment, as defined by European LeukemiaNet criteria (22). The complete hematological response (CHR) was defined as hematocrit < 45% with no phlebotomy in the past 3 months, platelet count < 400 × 10⁹/L, and leucocyte count < 10 × 10⁹/L. The secondary endpoints were molecular response and safety at 48 weeks of treatment. In this study, the JAK2V617F mutation allele burden was used to evaluate the molecular response. Complete molecular remission (CMR) (based solely on the assessment of a JAK2V617F mutation allele burden) required undetectable JAK2V617F, whereas partial molecular remission (PMR) was defined as a reduction of more than or equal to 50% in patients with less than 50% mutant allele burden, or a reduction more than or equal to 25% in patients with more than 50% mutant allele burden (22). All patients gave informed consent to the publication of the data. The project was approved by the ethics committee of the First Affiliated Hospital of Soochow University (No. 089).

### Statistical Analysis

Responses and clinical data were analyzed with descriptive statistics. Pearson’s chi-square test, continuous calibration chi-square test, and Fisher’s exact test were used to compare categorical variable in different groups. The *t* test and one-way analysis of variance were used to compare continuous variables in different groups. When the dependent variable was categorical, univariate or multivariate logistic regression analysis was used for correlation analysis. When the dependent variable was continuous, univariate or multivariate linear regression was used for correlation analysis. GraphPad Prism 8.0 and SPSS 20.0 software were used for all analyses. *P* value < 0.05 denoted statistical significance.

## Results

### Patient Characteristics

A total of 44 patients (19 with PV and 25 with ET) received Peg-IFN-a-2b in the treatment cohort between November 2017 and October 2019, including 5 patients receiving first-line treatment and 39 receiving second-line treatment. 16 patients were treated with cytoreductive therapy for less than 3 months, of which 2 were treated with short-acting IFN combined with HU, and 14 were treated with HU monotherapy. In this study, the median follow-up time was 15.0 months (rang 2.6–43.0 months), and the median age of the patients was 51 years (range, 20–67 years); 47.6% (20/42) patients had splenomegaly, 81% (34/42) had previously received HU treatment, 11.9% (5/42) had a history of thrombosis, and 57.14% (24/42) had various degrees of myelofibrosis. The interval from the first diagnosis to the treatment was 4.6 months (range 0–157.5 months). Among patients with PV, 76.5% (13/17) were of low risk and 23.5% (4/17) were of high risk, while among patients with ET, 52% (13/25) were of very low risk, 16% (4/25) were of low risk, 28% (7/25) were of intermediate risk, and 4% (1/25) were of high risk according to 2018 NCCN (23). Thirty-four patients (34/44, 77.27%) tested positive for JAK2V617F, and four patients (4/25, 9.10%) tested positive for calreticulin (CALR). Five (5/25, 20%) were triple negative for JAK2, CALR, and MPL in patients with ET, while only one was triple negative in patients with PV. Since the triple-negative PV patient had previously received HU and Phlebotomy, we did not detect serum erythropoietin level before treatment. Baseline characteristics of patients in the entire cohort were outlined in **Table 1**. Details of the 6 triple-negative patients were summarized in **Table 2**.

**Table 1:** Baseline characteristics of the entire cohort.

	PV	ET	Total	*P* value
Male (%)	63.20 (12/19)	32.00 (8/25)	45.50 (20/44)	**0.040**
Female (%)	36.80 (7/19)	68 (17/25)	54.50 (24/44)	**0.040**
Age (year)				
Median (range)	53 (32–67)	50 (20–67)	51 (20–67)	0.061
Splenomegaly (%)	57.90 (11/19)	39.10 (9/23)	47.60 (20/42)	0.226
Hydroxyurea pretreated (%)	94.10 (16/17)	72.00 (18/25)	81.00 (34/42)	0.163
Previous thromboembolic event (%)	23.50 (4/17)	4.00 (1/25)	11.90 (5/42)	0.152
Disease duration in months since diagnosis, median (range)	2.50 (0.20–140.00)	7.00 (0.00–157.50)	4.60 (0.00–157.50)	0.743
Gene mutation				
JAK2 (%)	94.74 (18/19)	64.00 (16/25)	77.27 (34/44)	**0.041**
CALR (%)	0	16.00 (4/25)	9.10 (4/44)	–
MPL (%)	0	0	0	–
Triple negative (%)	5.26 (1/19)	20.00 (5/25)	13.63 (6/44)	0.333
Risk stratification				
Very low risk (%)	–	52.00 (13/25)	–	–
Low risk (%)	76.50 (13/17)	16.00 (4/25)	–	–
Intermediate risk (%)	–	28.00 (7/25)	–	–
High risk (%)	23.50 (4/17)	4.00 (1/21)	–	–
Positive status for JAK2 Val617Phe mutation				
Mean allele burden (%)	54.02 ± 23.90	28.01 ± 14.28	42.20 ± 23.79	**0.033**
Mean hematocrit (%)	52.34 ± 7.12	38.98 ± 9.25	44.87 ± 10.66	0.467
Mean hemoglobin (g/L)	171.58 ± 21.84	135.17 ± 14.61	151.26 ± 25.61	**0.033**
Mean platelet count (10⁹/L)	425.21 ± 223.89	750.98 ± 315.93	607.03 ± 320.84	0.410
Mean leucocyte count (10⁹/L)	11.61 ± 5.65	9.08 ± 4.02	10.20 ± 4.91	0.163
Myelofibrosis				
0 grade (%)	61.11 (11/18)	29.17 (7/24)	42.86 (18/42)	**0.038**
1 grade (%)	38.89 (7/18)	66.67 (16/24)	54.76 (23/42)	0.073
2 grade (%)	0	4.17 (1/24)	2.38 (1/42)	–

“-” Not done. Bold values indicate the following: 1. The proportion of male patients in PV patients was significantly higher than that in ET patients, while the proportion of female patients in PV patients was significantly lower than that in ET patients (p = 0.040). 2. The proportion of JAK2 mutation in PV patients was significantly higher than that in ET patients (p = 0.041). 3. Before treatment, JAK2 mutation burden and hemoglobin in PV patients were significantly higher than those in ET patients (p = 0.033 and p = 0.033, respectively). 4. Before treatment, the proportion of PV patients without myelofibrosis was significantly higher than that of ET patients without myelofibrosis (p = 0.038).

**Table 2:** Detailed characteristics of 6 patients with triple negative MPNs before treatment.

NO.	Age(years)	Gender	Diagnosis	Previous Treatment	Thrombosis	Cardiovascular Risk Factors	WBC (×109/L)	HB (g/L)	HCT (%)	PLT (×109/L)	LDH (U/L)	Bone Marrow Biopsy	CHR at 48 Weeks of Treatment
01	55	Male	PV	HU;Phlebo-tomy	No	Yes	5.14	170	47.3	171	236.5	Hypercellularity with prominent erythroid proliferation; MF 0.	No (CHR at 12 weeks of treatment)
02	42	Female	ET	IFN;HU; Aspirin	No	No	NA	NA	NA	NA	151	Hypercellularity; Megakaryocytosis with hyperlobulated nuclei; MF 1.	Yes
03	40	Female	ET	IFN;HU	No	No	7.41	130	39.5	1135	204.7	Hypercellularity; Megakaryocytosis with hyperlobulated nuclei; MF 1.	No
04	20	Female	ET	Aspirin	No	No	5.7	132	NA	1430.3	219	Hypercellularity; Megakaryocytosis with hyperlobulated nuclei; MF 0.	No
05	47	Female	ET	IFN;HU; Aspirin	No	No	7.22	124	37.6	691	151	Hypercellularity; Megakaryocytosis with hyperlobulated nuclei; MF 1.	Yes
06	56	Female	ET	Aspirin	No	No	8.91	119	35.4	533	NA	Hypercellularity; Megakaryocytosis with hyperlobulated nuclei; MF 1.	NA (Interruption at 26 weeks of treatment)

WBC, white blood cells; HB, hemoglobin; PLT, platelets; HT, hematocrit; MF, myelofibrosis; LDH, lactate dehydrogenase; NA, not available.

### Efficacy in Control of Hemogram

**Figure 1:**
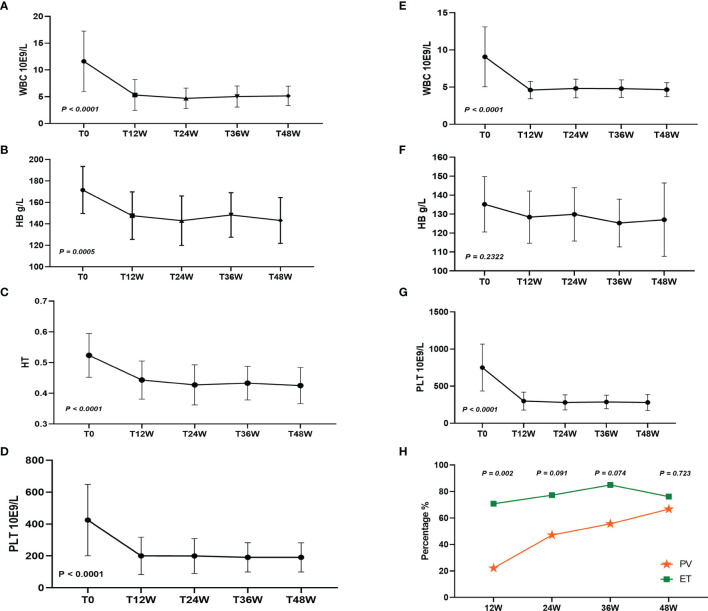
**(A–D)** White blood cell (WBC), hemoglobin (HB), hematocrit (HT), and platelets (PLT) changed after 48 weeks of Peg-IFN-a-2b treatment in patients with PV. **(E–G)** WBC, HB, and PLT changed after 48 weeks of Peg-IFN-a-2b treatment in patients with ET. **(H)** CHR at 12, 24, 36, and 48 weeks after treatment with Peg-IFN-a-2b, respectively.

After treatment with Peg-IFN-a-2b for 48 weeks, leukocytes, hemoglobin, hematocrit, and platelets in 19 patients with PV were significantly decreased (*P* < 0.0001, *P* = 0.0005, *P* < 0.0001, and *P* < 0.0001, respectively), and leukocytes and platelets in 25 patients with ET were significantly decreased (*P* < 0.0001 and *P* < 0.0001, respectively), while hemoglobin did not change significantly (*P* = 0.2322) (**Figures 1A–G**). After Peg-IFN-a-2b treatment for 12 weeks, among 42 evaluable patients, CHR was achieved in 50.00%, 22.22% (4/18) of patients with PV and 70.83% (17/24) of patients with ET, which was significantly higher than that of PV (*P* = 0.002). Among the 39 evaluable patients, a CHR rate was 64.10% (25/39) after 24 weeks of treatment with Peg-IFN-a-2b. Of these patients, CHR was achieved in 47.06% (8/17) of patients with PV and 77.27% (17/22) of patients with ET. No difference was found between the two groups (*P* = 0.091). After 36 weeks of treatment with Peg-IFN-a-2b, among the 38 patients with available data, CHR was achieved in 71.05% of patients, 55.56% (10/18) patients with PV and 85.00% (17/20) patients with ET. No difference was found in CHR between the two groups (*P* = 0.074). After Peg-IFN-a-2b treatment for 48 weeks, among 39 patients with available data, CHR was achieved in 71.79% of patients, 66.67% (12/18) patients with PV and 76.19% (16/21) patients with ET. No difference was found in CHR between the two groups (*P* = 0.723) (**Figure 1H**).

**Figure 2:**
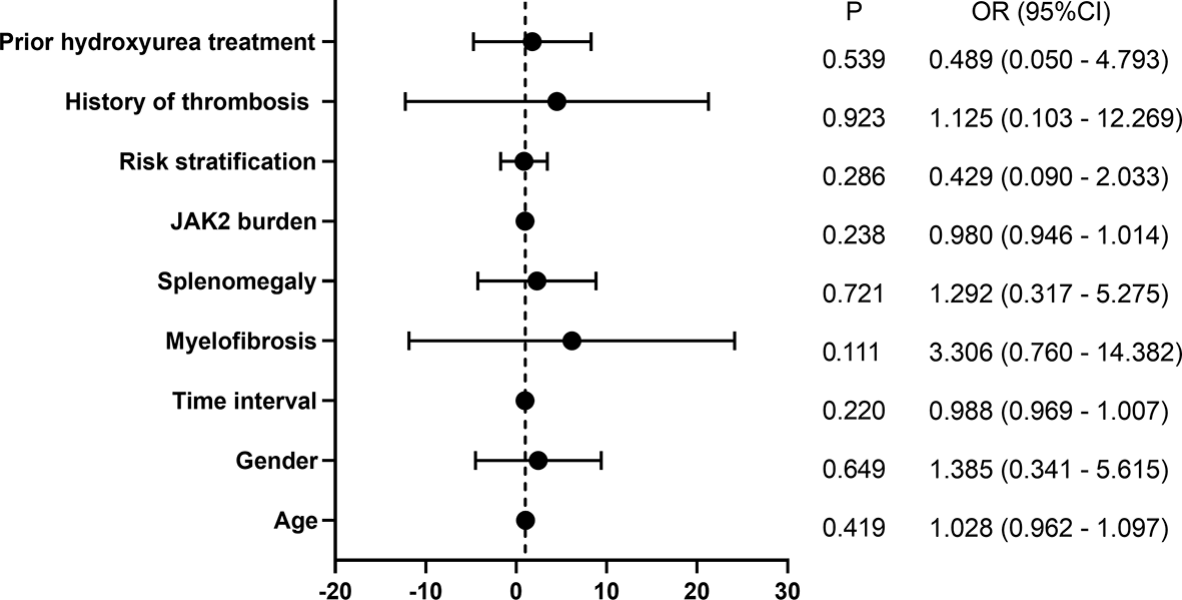
Univariate logistic regression analysis of CHR.

Of the 16 patients followed for more than 72 weeks, 10 patients gained CHR and 6 patients did not gain CHR at 48 weeks of treatment. At 72 weeks of treatment, 9 patients had sustained CHR, 2 patients achieved CHR, 1 patient lost hematologic response, and 4 still did not obtain CHR, suggesting that most patients maintained their hematologic response for a short period of time. Univariate logistic regression analysis showed that age, gender, interval from first diagnosis to the treatment, myelofibrosis, splenomegaly, JAK2 mutation burden, risk stratification, history of HU treatment, and history of thrombosis did not affect CHR (**Figure 2**).

### Changes in JAK2V617F Mutation Burden

**Figure 3:**
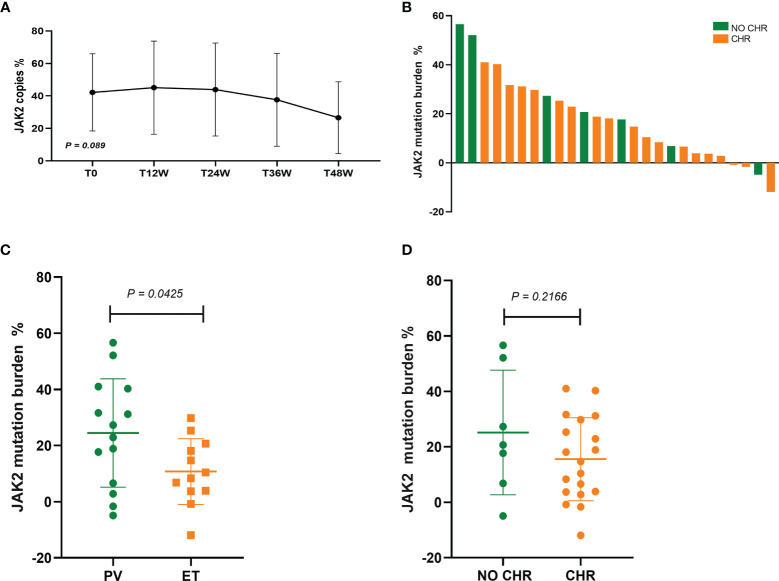
**(A)** Changes in JAK2 mutation burden at 12, 24, 36, and 48 weeks after Peg-IFN-a-2b treatment, respectively. **(B)** Absolute change in JAK2V617F mutation burden from baseline to 48 weeks of Peg-IFN-a-2b therapy. **(C)** Absolute change in the reduction of JAK2 mutation burden in patients with PV and ET after 48 weeks of Peg-IFN-a-2b therapy. **(D)** Absolute change in the reduction of JAK2 mutation burden in patients with and without CHR after 48 weeks of Peg-IFN-a-2b therapy.

In 34 patients with JAK2V617F-mutated PV and ET treated with Peg-IFN-a-2b, JAK2 mutation burden did not decrease significantly within 1 year (*P* = 0.089) (**Figure 3A**). Molecular evaluation was feasible in 26 patients at 48 weeks of treatment. No patients achieved CMR, and 43.48% patients achieved PMR (10/23, 3 cases of JAK2 mutation burden <10% before treatment were excluded). PMR was obtained in 38.46% (5/13) of patients with PV and 50.00% (5/10) of patients with ET, and no difference was found in molecular response between the two groups (*P* = 0.666). Compared with patients with ET, JAK2 mutation burden decreased significantly in patients with PV (*P* = 0.0425) (**Figure 3C**), which may be related to baseline JAK2 mutation burden prior to treatment. No difference in decrease in JAK2 burden was found between patients who achieved CHR and who did not (*P* = 0.2166) (**Figure 3D**), suggesting that the hematologic response was not associated with the reduction of JAK2 burden, which was consistent with previous findings (15) (**Figure 3**).

Molecular response data were available for 12 patients who were followed for more than 72 weeks, of whom 6 obtained PMR and 6 did not obtain molecular response at 48 weeks of treatment. During the follow-up, 5 (83.33%, 5/6) patients maintained the PMR status, 1 (16.67%, 1/6) lost molecular response, and 2 (33.33%, 2/6) gained PMR. The univariate regression analysis showed that age, sex, interval from initial diagnosis to this treatment, myelofibrosis, splenomegaly, history of HU treatment, thrombus history, and CHR did not affect the decrease in JAK2 mutation burden, while diagnosis and risk stratification did (*P* = 0.043 and *P* = 0.032, respectively). The multivariate regression analysis showed that neither diagnosis nor risk stratification affected the decrease in JAK2 mutation burden (*P* = 0.580 and *P* = 0.288, respectively).

### Reduction in the Spleen Size

Before treatment, 47.62% (20/42) patients, including 11 with PV and 9 with ET, had splenomegaly. Splenomegaly improved in 65.00% (13/20) of the patients at 48 weeks of treatment, including 10 with PV and 3 with ET. In 20 patients with splenomegaly, 10 patients achieved CHR and 5 patients achieved PMR at 48 weeks of treatment.

### Adverse Events

During the follow-up, 75% (33/44) of the patients discontinued or reduced Peg-IFN-a-2b, with a median time of 3.00 months (range, 0.70–35.90 months) from treatment initiation to Peg-IFN-a-2b discontinuance or reduction; 9.10% (4/44) patients discontinued treatment at 2.60, 4.70, 5.50, and 6.40 months due to adverse events, respectively; 52.27% (23/44) of the patients reduced the dosage due to adverse events; 4.55% (2/44) of the patients adjusted the treatment regimen due to poor efficacy; 9.10% (4/44) of patients reduced Peg-IFN-a-2b dosage after CHR. In general, Peg-IFN-a-2b was safe and well tolerated in most patients. The most common nonhematologic adverse events were fever, myalgia, itchy skin, elevated liver enzymes, and fatigue. Of 44 patients, 4 (9.10%) discontinued treatment due to drug-related toxicity (dyspnea and pneumonitis in 1 patient, increased aspartate aminotransferase and alanine aminotransferase in 2 patients, and severe fatigue in 1 patient). No thrombogenesis occurred when the patients were treated with Peg-IFN-a-2b. However, the results of Masarova and his colleagues showed a 1.22% annual incidence of thromboembolic events during treatment with Peg-IFN-a-2b (15), which may be due to the fact that IFN therapy increases prothrombotic biomarkers in patients with MPNs (24). Hematological toxicity manifested as grade 1–2 leukopenia, grade 1 thrombocytopenia, and grade 1 hemoglobin reduction, requiring no special treatment.

## Discussion

In this study, CHR was achieved in 66.67% of patients with PV and 76.19% of patients with ET after 48 weeks of Peg-IFNa-2b treatment, and 50% of patients obtained CHR at 3 months of treatment. At 48 weeks of treatment, molecular responses were obtained in 38.46% of patients with PV and 50% of patients with ET. A phase II clinical trial of Peg-IFN-a-2a also had similar results, and it showed that CHR was 70% in patients with PV and 76% in patients with ET, with most responses occurring within the first 3 months of the treatment, and the overall molecular responses of ET and PV were 38% and 54%, respectively (9). No difference in CHR was found between patients with ET and PV after 48 weeks of treatment in our study, while the data from the Myeloproliferative Disorders Research Consortium-111 study showed that CHR was higher in patients with ET than in those with PV after 12 months of second-line Peg-IFN-a-2a treatment (18). In a small cohort study from Taiwan, CHR was achieved in 62.5% of patients with MPNs treated with Peg-IFN-a-2b (25), while the present study showed a slightly higher CHR of 71.79%. The PROUD-PV and CONTINUATION-PV studies confirmed that the 1-year CHR was 53% and the 3-year CHR was 71% in PV patients treated with ropeginterferon alfa-2b (19), suggesting that CHR may increase with the extension of treatment time. It is worth noting that ropeginterferon alfa-2b in this study is a monopegylated IFN-a developed for treating MPNs. In contrast to other Peg-IFN-a compounds, ropeginterferon alfa-2b consists of a single positional isomer resulting in an extended elimination half-life, enabling less frequent dosing and improved tolerability (19). Our study only showed that CHR of PV patients treated with Peg-IFN-a-2b increased within 1 year, which was limited by the short follow-up time. In another phase II multicenter study of PV, CHR was 94.6% after 12 months of Peg-IFN-a-2a treatment, and all patients maintained CHR during follow-up (median of 31.4 months) (12). These results were consistent with those of the present study, which showed that 90% (9/10) of patients maintained CHR and 10% (1/10) lost to hematologic response at follow-up. However, these findings appeared to conflict with the results of Masarova and colleagues, which showed that 80% of patients with PV and ET treated with Peg-IFN-a-2a gained CHR, with a mean duration of 66 months, and only 39% maintained CHR at the last follow-up (median follow-up of 83 months), indicating that nearly half of patients lost to hematologic response 5 years after treatment (15). Their data also showed that the duration of PMR was 49 months, and that most patients who achieved PMR lost the optimal response during the follow-up (15). The present study confirmed that 83.3% (5/6) of patients maintained PMR and 16.7% (1/6) of the patients lost molecular response during the follow-up. The difference between the results of this study and previous data may be due to the fact that the median follow up time in this study was not long enough. The present study showed that no patient achieved a CMR after 12 months of Peg-IFN-a-2b therapy, which may be due to the gradual realization of molecular responses over time, with a median time to response of 24 months according to previous studies (15). Recently, interim analysis of a multicentre, randomised phase 2 clinical trial on low-risk patients with PV provided evidence that Peg-IFN-a-2b in addition to phlebotomy and low-dose aspirin is superior to phlebotomy and low-dose aspirin alone in keeping low-risk patients with PV on haematocrit target (26). This evidence is sufficient to support the fact that MPNs therapy, especially for low-risk patients, can not be limited to conventional therapy, and Peg-IFN should be actively promoted.

Elimination of JAK2V617F-mutated long-term HSCs was required to achieve long-term disease remission or cure. However, only a very small number of patients who receive Peg-IFN achieve CMR, and the long-term efficacy of Peg-IFN is of worrisome (12, 15). Thus, therapies that could overcome the embarrassment were urgently needed. JAK inhibitor ruxolitinib is a second-line drug commonly used in treating PV or ET, but it fails to demonstrate superiority over best supportive care in patients with HU-resistant or intolerant ET (27). Studies have shown that ruxolitinib mainly acts on hematopoietic progenitor cells and can relieve constitutional symptoms of patients with MPNs, but has limited capacity to reduce JAK2V617 burden. IFN directly affects JAK2V617F-mutated HSCs, causing DNA damage and intracellular reactive oxygen species, eventually leading to the elimination of JAK2V617F-mutated HSCs. These findings provide a theoretical basis for multidrug combination (28). Clinical trial data from a phase II study of combination therapy with Peg-IFN and ruxolitinib in MPNs patients who are resistant and/or intolerant to IFN monotherapy have confirmed that CHR in patients with PV was 44% after 12 months of treatment (29). However, the discontinuation rate was as high as 20% due to treatment-related toxicity (29). Published studies confirmed that ruxolitinib caused DNA repair defects and sensitized MPNs stem and progenitor cells to poly-ADP-ribose polymerase (PARP) inhibitors olaparib and BMN673, and HU induced DNA damage; therefore, ruxolitinib and olaparib plus or minus HU were very effective *in vivo* against JAK2 (V617F)^+^ murine MPN–like disease (30). Their clinical antitumor activity and safety need to be further verified. At present, the initial dose is 45-450µg per week in clinical trials of Peg-IFN for MPNs, and the discontinuation rate varied due to treatment-related toxicity (12, 15, 18). This study showed that patients received Peg-IFN-a-2b at a dose of 180 µg weekly, and 9.1% of patients discontinued treatment due to adverse events, which was lower than that of the published data. These data imply that the current optimal drug dose for peginterferon is still empirical, and the balance between optimal efficacy and optimal tolerability deserves further exploration.

In brief, the treatment of MPNs is changing, and Peg-IFN has been proved to be an effective options for MPNs regardless of clinical trials and the real life. For patients with poor response to Peg-IFN monotherapy, Peg-IFN-based combination therapy and genetic landscape analysis can be considered.

## Data Availability Statement

The original contributions presented in the study are included in the article/supplementary material. Further inquiries can be directed to the corresponding author.

## Ethics Statement

The studies involving human participants were reviewed and approved by the Ethics Committee of The First Affiliated Hospital of Soochow University. Written informed consent to participate in this study was provided by the participants’ legal guardian/next of kin.

## Author Contributions

YS wrote the manuscript. SC guided the treatment of cases. YC, JC, MZ, QW, and JP performed the research and analyzed the data. All authors contributed to the article and approved the submitted version.

## Funding

This study was supported by grant from the National Key R&D Program of China (2019YFA0111000), the National Natural Science Foundation of China (81900130, 81970136, 81970142, 82000132, 8217011130, 8210010924), the Natural Science Foundation of the Jiangsu Higher Education Institution of China (18KJA320005, 18KJB320019), the Natural Science Foundation of Jiangsu Province (BK20190180, BE2018652), Priority Academic Program Development of Jiangsu Higher Education Institution, the Innovation Capability Development Project of Jiangsu Province (BM215004), the Translational Research Grant of NCRCH (2020WSB03, 2020WSB11, 2020WSB13), the Open Project of Jiangsu Biobank of Clinical Resources (SBK202003001, SBK202003003).

## Conflict of Interest

The authors declare that the research was conducted in the absence of any commercial or financial relationships that could be construed as a potential conflict of interest.

## Publisher’s Note

All claims expressed in this article are solely those of the authors and do not necessarily represent those of their affiliated organizations, or those of the publisher, the editors and the reviewers. Any product that may be evaluated in this article, or claim that may be made by its manufacturer, is not guaranteed or endorsed by the publisher.
